# Design, Simulation, and Fabrication of a Copper–Chrome-Based Glass Heater Integrated into a PMMA Microfluidic System

**DOI:** 10.3390/mi12091067

**Published:** 2021-09-02

**Authors:** Santiago Tovar, Cesar A. Hernández, Johann F. Osma

**Affiliations:** CMUA, Department of Electrical and Electronic Engineering, Universidad de los Andes, Carrera, 1E # 19A-40, Bogotá 111711, Colombia; s.tovar@uniandes.edu.co (S.T.); ca.hernandez11@uniandes.edu.co (C.A.H.)

**Keywords:** microheater, microsystem, electrothermal systems, thermal characterization, hysteresis, physical vapor deposition (PVD), Polymethylmethacrylate (PMMA)

## Abstract

In this paper, the development of a copper–chrome-based glass microheater and its integration into a Polymethylmethacrylate (PMMA) microfluidic system are presented. The process highlights the importance of an appropriate characterization, taking advantage of computer-simulated physical methods in the heat transfer process. The presented system architecture allows the integration for the development of a thermal flow sensor, in which the fluid flows through a 1 mm width × 1 mm length microchannel across a 5 mm width × 13 mm length heating surface. Using an electrothermal analysis, based on a simulation and design process, the surface heating behavior curve was analyzed to choose a heating reference point, primarily used to control the temperature point within the fluidic microsystem. The heater was characterized using the theory of electrical instrumentation, with a 7.22% error for the heating characterization and a 5.42% error for the power consumption, measured at 0.69 W at a temperature of 70 °C. Further tests, at a temperature of 115 °C, were used to observe the effects of the heat transfer through convection on the fluid and the heater surface for different flow rates, which can be used for the development of thermal flowmeters using the configuration presented in this work.

## 1. Introduction

Heating in microsystems, referred to as microheaters, has a fundamental role in the operation of different devices, such as thermal flow sensors [1], microelectromechanical systems (MEMS) gas sensors [2,3,4,5], liquid petroleum sensing (LPG) [6], trace detection of explosives [7], thermal electric generation on microchannels [8], and temperature control of environment for chemical and biological processes at small scales [9,10]. The temperature control of microsystem devices can be achieved using macroscale equipment, such as incubators and surface heaters. This type of equipment usually consumes a considerable amount of power and does not allow performing parallel system-specific adjustments. Moreover, these macroscopic temperature controls, if precise, usually have slow time responses [11].

The design of temperature controls directly integrated into the microsystem architecture [12] is an auspicious alternative that has been credited for biological monitoring microsystems [10,13], polymerase chain reaction integrated devices [14], protein characterization and sensing [15], and supported bilayer lipid membrane-based biosensors [16], among others. These microsystems employ a thin film of deposited metal as an electrically resistive heating element, as described by Joule’s law. The importance of having direct contact between the heater and the system relies on having control and certainty about the conditions of the elements immersed in the microsystem, due to a resistance reduction in the heating transference [17], in addition to having the possibility to include thermal sensors using similar thin-film technologies within an integrated system [18].

An adequate characterization process on a heating system gives more precise results in the operating temperature [19,20,21]. For instance, in the works published by Scorzoni [20], Byers [21], and Tiwari [22], different designs for printed microheaters were presented, using COMSOL for modeling temperature and voltage distributions, highlighting the advantages of implementing electrothermal simulations to compare and analyze the characterization process on a heating system.

Similar approaches are currently used for fabricating microheaters, with diverse fabrication methods, both combined or not combined, such as physical vapor deposition (PVD) deposition methods [6,10,23,24,25], photolithography with digital light processing [23], and laser beam techniques [25]. Despite all the available techniques, research in emerging countries requires a more cost-effective approach.

On that basis, a heater was developed through a PVD deposition method and designed with a serpentine shape. All previous features mentioned gave optimal temperature requirements, as well as a good heat concentration on the device. Furthermore, it was characterized in detail, interpreting the hysteresis behavior, and was compared with electrothermal COMSOL simulations. The results were analyzed to choose a heating reference point, providing precise temperature results. The fabricated heater was integrated into a PMMA microsystem. This integration seeks to find an alternative to the use of cleanroom facilities, taking advantage of readily available equipment, such as a laser cutting machine, reducing fabrication costs and labor for research activities, thus offering a desired characteristic for research in emerging countries. From the reference heating point, a control current system was designed and implemented to maintain the temperature within working boundaries at the microfluidic system. The electrothermal results supported the simulation, and it granted a rapid, sustained, and validated low-power thermal control, achieving a thermal characterization in which a high-resolution heat profile distribution phenomenon, at different flow rates, was observed by thermal imaging, backing up the possibility to integrate thermal flow sensors, with the advantage of having the flexibility of being coupled to different systems, as well as having optical transparency and repeatability in its fabrication process.

## 2. Materials and Methods

### 2.1. Materials, Equipment, and Software

Polymethylmethacrylate (PMMA) sheets with a thickness of 2 mm and 3 mm were bought from Diacrílicos J.D. (Colombia, Bogotá). Glass slides with dimensions of 25.4 mm × 7.2 mm × 1.2 mm were purchased from vendor Sail Brand (Yancheng, China). Chrome pieces, Cr, purity of 99.95%, with a size range between 0.8 mm to 6 mm, and copper pellets, purity 99.99%, with a diameter of 6.35 mm and a length of 6.35 mm were acquired from Kurt J. Lesker Company (Clairton, PA, USA). Analytical-grade methylene chloride was used. The physical vapor deposition (PVD) process was performed using thermal evaporator Edwards E306 (Moorfield Nanotechnology Limited, Knutsford, Cheshire, UK) in a cleanroom facility environment. Spin coating was performed using a SPIN150 spin coater (SPS Europe B.V., Putten, The Netherlands). UV photolithography was performed using a Karl-Suss MJB-3 Aligner (SÜSS MicroTec SE, Garching, Germany). Positive photoresist MICROPOSIT™ SC™ 1827 (SC-1827) and developer MICROPOSIT™ MF™ 319 (MF-319 developer) were purchased from Rohm and Haas Electronic Materials LLC (Marlborough, MA, USA). Baker PRS-1000 was used as stripper for lift-off processes and was purchased from Avantor (Radnor, PA, USA). PMMA slides were fabricated with a laser cutting machine Trotec^®^ SPEEDY 100 (Trotec Laser GmbH, Marchtrenk, Austria). Transparent double-sided tape TESA, with a thickness of 205 um (Tesa SE, Hamburg, Germany), was used for sealing of the microdevices. Special glue UHU HART (UHU GmbH & Co. KG, Bühl, Germany) was used for gluing of coupling connectors.

The design of the printed circuit board (PCB) was elaborated using EAGLE 8.3.2 PCB Design Software (Autodesk, San Rafael, CA, USA). The design of the PMMA microsystems was developed using AutoCAD 2020 Design Software (Autodesk, San Rafael, CA, USA). A thermoelectric analysis simulation was implemented in COMSOL Multiphysics 5.3 (COMSOL, Inc., Burlington, MA, USA). Supply and measurements for the microsystem characterization process were carried out with variable dual voltage source HAMEG^TM^ HM7042 (HAMEG Instruments GmbH, Mainhausen, Germany), digital multimeter PeakTech Multifunction Tester^®^ 3725 (PeakTech Prüf und Messtechnik GmbH, Ahrensburg, Germany), and thermal camera Panasonic AMG8833 (Panasonic Industrial Devices Company, Newark, NJ, USA).

The simulation of the temperature control system with the current source (IC) was developed in LTSpiceXVII of Analog Devices (Analog Devices, Inc. (ADI), Norwood, MA, USA). For the integration of the IC, 2N2222 transistors from Microsemi (Microchip Technology Inc, AZ, USA), a 15 Ω metal film resistor 0.5 W 1% (KOA Speer Electronics Inc., Bradford, PA, USA), and an adjustable trimmer 100 Ω BOURNS 3296 W-1-101_LF (Bourns Inc., Riverside, CA, USA) were used. The measurements of the microsystem heater were performed with thermographic camera Keysight U5855A JP54270688 (Keysight Technologies, Santa Rosa, CA, USA) and syringe infusion pump Medcaptain MP-30 (Medcaptain Medical Technology Co. Ltd., Guangdong Sheng, China). To perform the leak test and report the thermographic camera results, transparent water-based olive green (956) dye made with pigments and with a matte finish ROSETA (ROSETA INDUSTRIAL LTDA, Colombia) was used.

### 2.2. Copper–Chrome-Based Glass Heater

**Figure 1:**
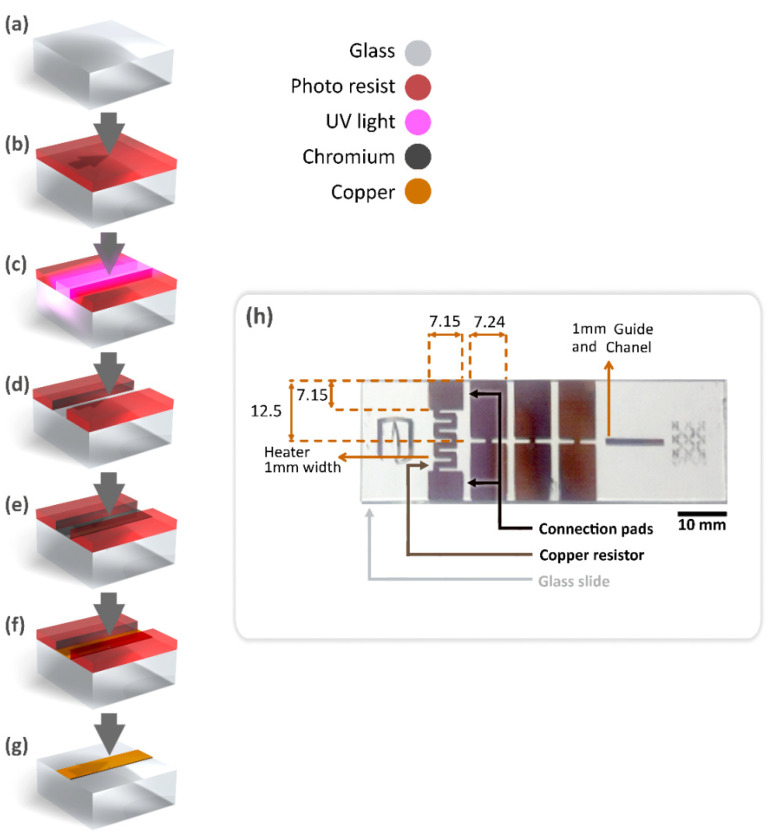
Fabrication process of the copper–chrome-based glass heater: (**a**) clean glass surface; (**b**) photoresist-covered glass; (**c**) UV light exposure; (**d**) photoresist development; (**e**) chrome deposition; (**f**) copper deposition; (**g**) final heater structure on glass after stripping off photoresist; (**h**) Photograph of the completed copper–chrome-based glass heater with dimensions.

For the fabrication of the copper–chrome-based glass heater, a photolithographic mask was designed (Figure 1) featuring two connection pads, each of 7.15 mm × 7.15 mm and a 1 mm width path to outline the heater trace, with a total longitude of 37 mm, in the shape of a rectangular serpentine with 1 mm space between each lap and a 5 mm width × 13 mm length. Additionally, the photolithographic mask included the design of three extra connection pads, designed to allow the future integration of temperature sensors into the microsystem.

The heater was built upon a regular glass slide (Figure 1). First, the glass slides (Figure 1a) were spin-coated (Figure 1b) with SC-1827 photoresist at 5000 rpm for 1 min and precured at 100 °C for 50 s on a hotplate. Coated glass slides were exposed to UV light using a photolithographic mask (Figure 1c), developed (Figure 1d), and exposed directly to UV light for 1 min. Then, a 15 nm chrome layer was deposited by physical vapor deposition (Figure 1e), for a 10 mg chrome evaporation, achieved using an electrically heated tungsten filament. A vacuum pressure of 7 × 10^−6^ mbar and an evaporation rate of 1 nm/s were established. Afterward, a 250 mg copper evaporation was performed (Figure 1f), using the same evaporation parameters. As a result, metallic layers with a 115 nm thickness compounded by a 15 nm chrome nanolayer and a 100 nm copper nanolayer (Figure 1g) were achieved. The fabricated heater is shown in Figure 1h.

### 2.3. Microsystem Assembly Process

**Figure 2:**
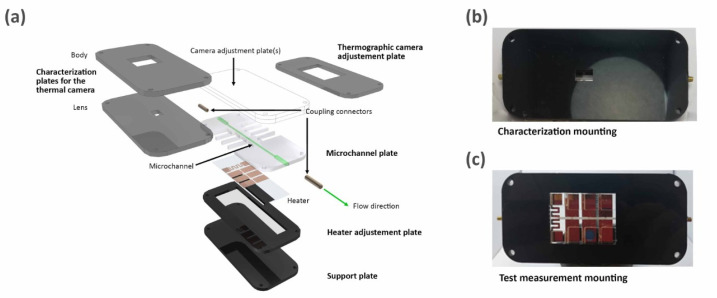
(**a**) Microsystem building diagram and final mounting photographs of (**b**) test measurement and (**c**) characterization.

The manufacturing technique for the PMMA microsystems was previously described by our group [13,26]. This technique is based on a series of characterized 1 mm cuttings and engravings with a laser-cutting machine on PMMA layers [27]. To assemble the PMMA plates and the heater, double-sided tape was used, which was also previously cut using the laser-cutting machine. The final layered plate design for the PMMA microsystem is depicted in the Figure 2. The microchannel plate contains a 1 mm engraving and spaces corresponding to the coupling pads used for wiring, and it was designed to adjust the current source to supply energy to the heater resistor (Figure 2a). The next plate, i.e., the heater adjustment plate, was dimensionally adjusted to the heater. The support plate was used in the prototype base with a second glass slide to give it the same thickness to the area reserved for the heater placement (2 mm) (Figure 2a).

The thermographic camera adjustment plate (Figure 2c) was glued to the top layer of the microchannel plate, giving the test measurements a wider visual angle than that provided by the characterization plate for the thermal camera (lens) and characterization plate for the thermal camera (body) (Figure 2b), which were glued to the top layer of the microchannel plate, wherein another layer was the microchannel coupled with the heater. In the final assembly of the microsystem (Figure 2a), the characterization mounting (Figure 2b), using the two characterization plates (lens and body), was employed for the test measurements using the thermographic camera, while, for the test measurement mounting, only the characterization plate for the thermal camera was used (Figure 2c). It can be noted that light transmission is avoided in the structure outside of the window area, which allows carrying out the correct data and photo recording with both cameras.

Lastly, to determine leaks and performance of the applied microflow, the complete microsystem was evaluated using a vacuum test with two empty syringes connected in both microsystem coupling connectors and a series pressure configuration of 12 mL/h with the syringe infusion pump, where air was used first as fluid, followed by water with transparent water-based green dye (concentration of ~3%), to observe the flow transition within the system.

### 2.4. Electrothermal Characterization

The characterization and calibration curves are an explicit relationship between a stimulus (*x*-axis, voltage) and its response (*y*-axis, temperature). These curves were developed including the system hysteresis, defined by a heating curve (up) and a cooling curve (down). A hysteresis curve, given by the theory of electrical instrumentation [28], can be obtained using the following equation:

(1) $$H=\left|Y_{1}-Y_{2}\right|,$$

where *Y*_1_ is the resulting response of the heating curve and *Y*_2_ is the resulting response of the cooling curve. These characterization curves were elaborated using the thermal camera, suited with an in-house interface, to obtain a resolution of 8 × 8 pixels with a size of 2.5 × 2.5 mm each. These measurements of voltage and temperature were developed with a variation of 0.1 V by step voltage variation. Furthermore, the current was measured within the characterization process to obtain the power consumption on the basis of the electrothermal characterization curve. The determined time of stabilization to measure each step was 10 s.

### 2.5. Electrothermal Simulation

The thermoelectric simulations were used to predict and compare the thermal behavior of the heater in response to a provided voltage, according to the electrothermal characterization. These simulations were defined for the computational model with similar properties and the same dimensions of the designed heater, as shown in Table 1. Moreover, the mesh size used in the model simulation was extra fine and was refined using the adaptive mesh refinement tool in COMSOL Multiphysics^TM^.

The power consumption was simulated on the basis of the electrothermal characterization curve using the same properties, which enabled a comparison of the power behavior in the fabricated microheater.

### 2.6. Point Thermal Characterization of the Fluid

**Figure 3:**
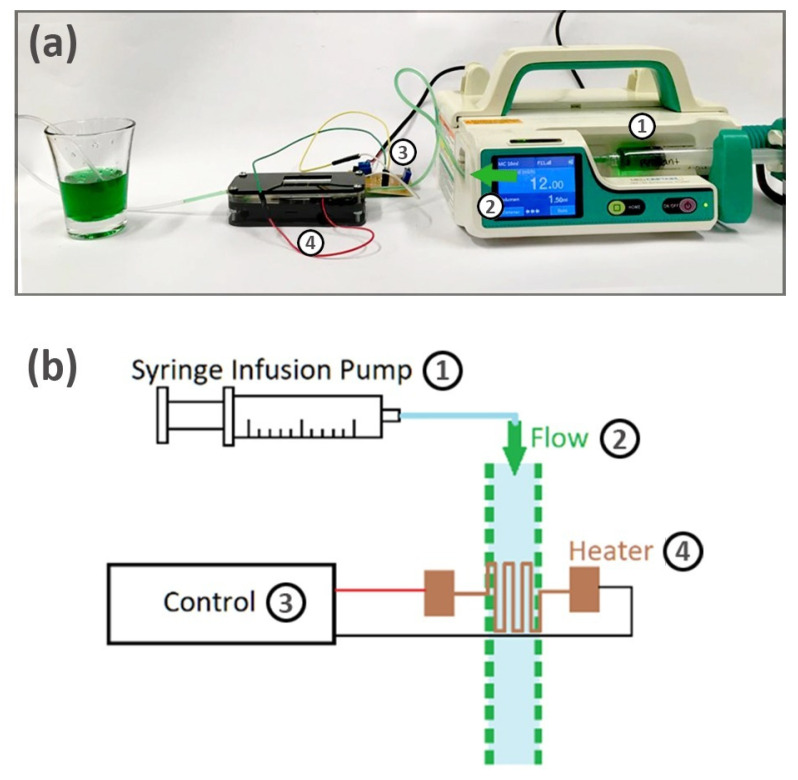
Test assembly with a thermographic camera: (**a**) schematic; (**b**) photograph.

For the point thermal characterization, the microsystem was connected to the heating control system, via the coupling wires, and to a syringe infusion pump, via the catheter, with a flow rate of 12 mL/h, as depicted in Figure 3. The fluid used in the system was the same as the fluid applied in the leak test, i.e., water with transparent water-based green dye (concentration of ~3%), to observe the flow transition within the system acquired by the thermographic camera, which was used with a ~15 cm distance to the microsystem, focusing on the heater as a temperature reference in all the image captures.

For the final test, the temperature was increased to 115 °C by maintaining a constant applied current, allowing an observation of the effects of heat convection in the heater surface due to the liquid flow as the temperature gradient between the heater and the liquid was increased, thus showing a temperature decrease through the flow direction line. The microsystem temperature profile was observed for flow rates of 5 mL/h, 10 mL/h, 15 mL/h, and 20 mL/h, to inspect the heat profile distribution phenomenon, which is produced due to the thermal advection and convection principle, whereby, as flow increases, convective heat loss increases from the heated element [20].

## 3. Results

### 3.1. Theoretical and Practical Resistance Analysis

Table 2 shows the common theoretical calculations for the resistance of the copper–chrome-based glass heater. The heater resultant resistance was the sum of both the pads and heater resistance, whereas this resistance was calculated using the parallel resistance value between the copper and the chrome structures. As a result, the theoretical resistance of the heater was 4.80 Ω.

Practical resultant resistance was measured for five identical heaters, produced as detailed in the methods. Each heater was measured four times, with a mean result of 9.11 Ω and variance of 0.17 Ω. Additionally, the microsystem coupling wire resistance was measured, with a mean value of 1.4 Ω and a variance of 0.22 Ω.

### 3.2. Thermal Camera Characterization

**Figure 4:**
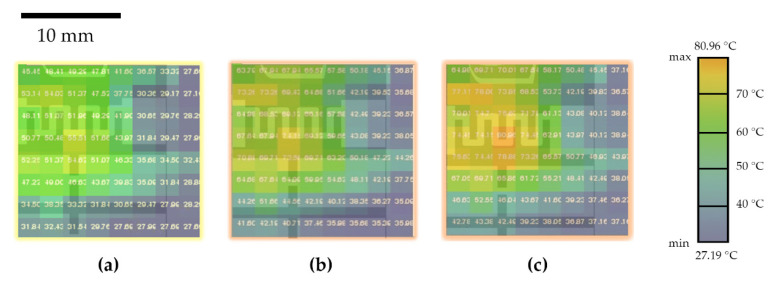
Image capture of the thermal camera with an applied voltage of (**a**) 2 V (peak temperature of 55.51 °C), (**b**) 2.5 V (peak temperature of 74.15 °C), and (**c**) 3 V (peak temperature of 80.96 °C).

Figure 4 shows images captured during the characterization process; each value was obtained by recording the maximum value of temperature, measured in the capture after a stabilization time of 10 s. The concentrated distribution of heat within the heater zone can be observed in Figure 4, and the temperature values correlated to the voltage provided, whereby 2 V corresponded to 55.51 °C (Figure 4a) at its peak, 2.5 V corresponded to 74.15 °C (Figure 4b), and 3 V corresponded to 80.96 °C (Figure 4c). A clear heat concentration in the neighboring area of the heater can be noted, thus confirming that the selected geometry of the heater allowed for a good heat concentration area.

**Figure 5:**
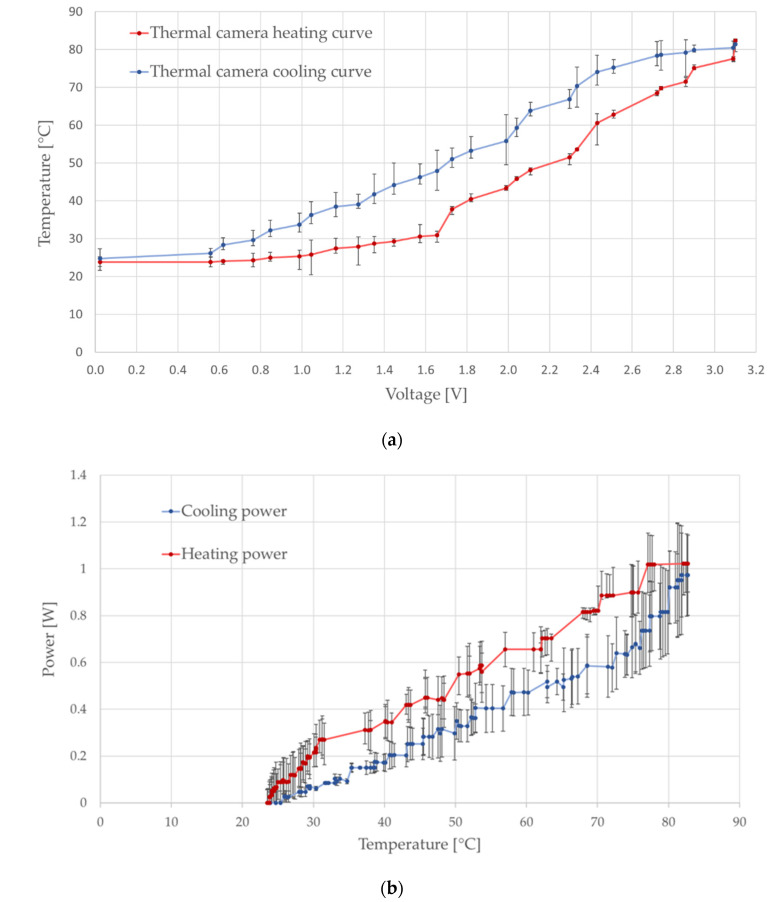
(**a**) System characterization for the thermal camera based in 0.1 V step changes; (**b**) Power consumption vs. temperature curve. The error bars show the standard deviation on each measurement point.

The resulting characterization of the heating system by the thermal camera is shown in Figure 5a, which illustrates the hysteresis behavior of the heater, described by the heating curve (red) and the cooling curve (blue). The heating curve showed a slow increase in temperature until an approximate voltage of 1.6 V was provided. After reaching this point, the temperature was observed to increase at a constant rate; this behavior can be seen in the heater from 1.8 V until reaching 3 V, indicating that, between these two conditions, the heater can be considered to linearly respond to the supplied voltage. In contrast, the cooling curve described a slow decrease in temperature until reaching 70 °C, which corresponds to 2.3 V when cooling down the microsystem. From this point, the cooling rate was constant until returning to room temperature; thus, the temperature response of the heater could be considered linear until reaching room temperature. This behavior reflects a rather slow heat dissipation of the heater microsystem materials, which can be used in systems that require maintaining a specific temperature over time. The power consumption was obtained for different focal temperature values (Figure 5b), which were measured using an infrared thermometer. It can be noted that, despite a good response of the heater to the applied voltage in terms of heating rate time, when compared to the cooling heat dissipation, the heater requires a relatively larger amount of power to produce heat; thus, if maintained at a constant voltage input, with no control of the consumed power, the system might tend to rise the working temperature. The error range observed in the power characterization can be attributed to a combination of the error range of voltage, current, and resist measured.

### 3.3. Heater Characterization Simulation

**Figure 6:**
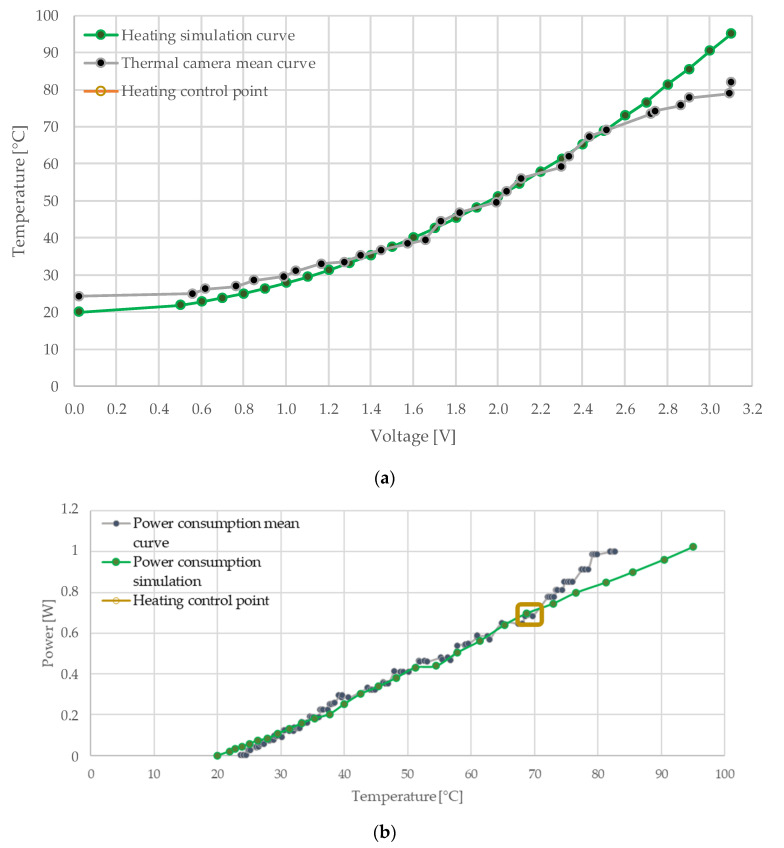
(**a**) Comparison system characterization between thermal camera mean curve and heating characterization curve behavior simulation based in 0.1 V step changes; (**b**) comparison between power consumption simulation based on resistance characterization curve and the mean measurement power consumption vs. temperature curve. Both comparisons have the heating control point highlighted.

The heating behavior characterization and power simulation curves are shown in Figure 6a,b. The heating behavior simulation values were compared with the respective thermal camera mean values, which yielded an estimate difference of 7.22% for the heating characterization. The power consumption simulation was implemented through a comparison between the temperature and the voltage simulation for the given resistance from the thermal camera characterization, with a 5.42% difference between both values. The acquired curves were used to establish a temperature setpoint, through the comparison between the electrothermal characterization and its simulation. This value was selected considering the temperature reference which possessed the least error range in the maximum point of sensibility, defined as the slope of the curve at a given point [17]. Specifically, an applied voltage of 2.47 V, which corresponds to a temperature of 70 ± 3 °C, was selected (Figure 6a). Additionally, a current intensity of 280 mA was measured for the heating system at this reference point. The obtained operation point was used as a constant reference for the heating temperature of the microsystem. The power within this heating control range provided in the simulation was 0.67 W, which is near to the power provided in the measurements, where the average power provided within the heating control range was 0.69 W (Figure 6b). According to the comparison provided in Figure 6a,b, the behavior observed in the measurements differed from that predicted in the simulation for temperatures above 70 °C, which were provided by an input voltage higher than 2.6 V; this difference can be explained by the ideal conditions of the simulation and the effects of the heat on the electric properties of the conductive layer.

**Figure 7:**
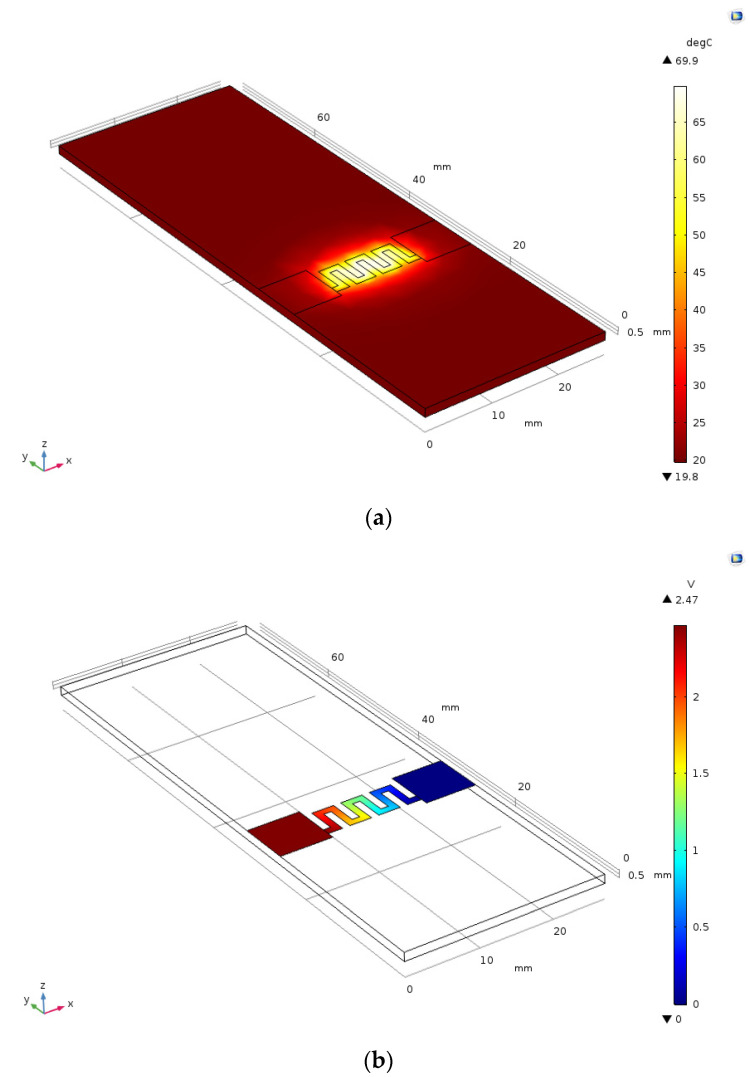
(**a**) Thermal distribution simulation (°C); (**b**) simulation of the electrical distribution (V), where it was immersed in water and air at room temperature, with the same heater sizes and similar properties to the heater, as shown in Table 2.

From the reference point selected, a thermal distribution simulation (Figure 7a) was performed, considering the same electrical and thermal characteristics as used for the characterization curve simulation and the same reference voltage provided to the heater, for which the voltage distribution simulation is shown in Figure 7b. The temperature distribution showed agreement with the thermal images captured during the characterization process, due to the thermic dissipation in the material surrounded by the designed rectangular serpentine geometry. Likewise, it can be observed that the provided voltage was evenly distributed throughout the heater geometry, which is explained by the divisions in the serpentine sections, where the lowest resistance is equally divided to focus the voltage proportionally, thus obtaining the temperature distribution that was previously observed. The power obtained in the simulation was 0.67 W, and the peak temperature in the thermal distribution simulation was 69.8 °C, which was adjusted to the thermal camera characterization and the heating behavior simulation.

### 3.4. Heating Control Circuit

The current source was designed and developed to set the current used at the heating point. The temperature control system design and its simulation were developed implementing 2N2222 transistors, to obtain a collector current of 300 mA, and, although there are market available mirrors and commercial encapsulated current sources, which are much more stable in their behavior, they were not able to provide this current intensity.

**Figure 8:**
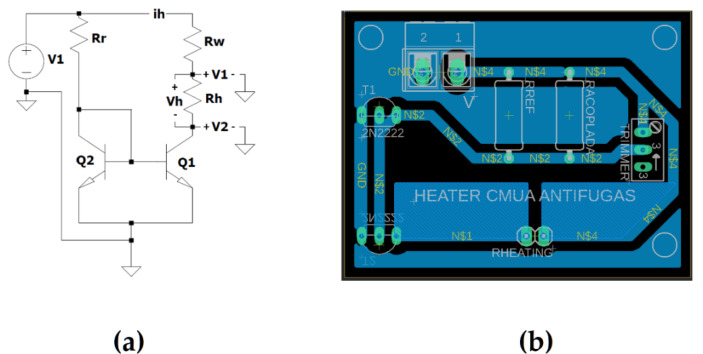
Heater circuit design: (**a**) simulation within reference point, where V1 (source voltage circuit) = 5 V, Rr (reference resistance) = 15 Ω, Rw (coupling wire resistance) = 1.4 Ω, Rh (homologated resistance heater) = 9.11 Ω, Q1 and Q2 (current mirror 2N2222 transistors), ih (current circuit) = 270.55 mA, V1 (voltage between homologated resistance heater and ground) = 4.62 V, V2 (collector–emitter Q1 voltage) = 2.16 V, Vh (resistance heater voltage, V2 − V1) = 2.47 V; (**b**) PCB design with the reference resistance and the 100 Ω parallel adjustment trimmer; (**b**) current source implemented in a PCB.

The electrical simulation for the circuit worked according to the Figure 8a with an output of 2.47 V in the heater resistance and a current of 270.5 mA, which represents the beginning of the chosen work area. Furthermore, as an indicator of good source performance [20,31], it was found to have an increase of 0.1 V and 10 mA due to the temperature increase in the circuit. Consequently, the calculated current source was implemented in a PCB, as observed in Figure 8b, providing the established power to the heater, in which the current was fixed by a reference resistance with a higher value than in the simulation, and which, in a parallel configuration with a trimmer, made it possible to lower the resistance to any specified value, adjusting the resistance value, if necessary, to change the reference resistance to the point at which the system would be worked.

For the evaluation of the current source, a current value of 280 mA and a voltage value of 2.49 V were observed, where the resistance of the heater was homologated by replacing the heater with a fixed resistance, also including the resistance of the cables and the pads that surround the heater rectangular serpentine shape at the time of stabilization. Then, the printed circuit board for this current source was assembled, where a voltage of 2.52 V and a current of 283 mA were achieved at its output by simulating the same heating resistance with the homologated resistance value. Lastly, the measurements were 2.53 V of output voltage and 279 mA of current with the heater and its coupling wires.

### 3.5. Thermal Characterization

**Figure 9:**
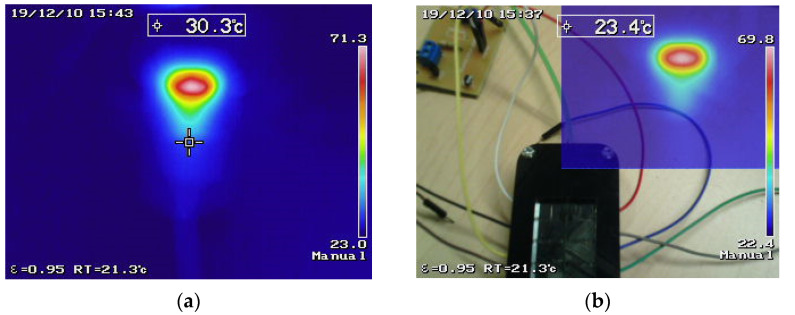
Thermographic camera captures with a controlled flow of 12 mL/h: (**a**) full-screen capture; (**b**) capture in a combined photo that shows the microsystem focalized area.

The image captures of the thermographic camera allowed observing the temperature distribution behavior in the microsystem when a flow was applied through the microchannel with the heater connected to the control circuit, as shown in Figure 9b. The reference temperature was 21.3 °C during the measurements, which is shown at the lower value of the scale in Figure 9. The maximum temperature achieved in the heater at the stabilization time was around 71.3 °C. In the thermal image capture, it can be observed that the heater produced a concentrated temperature gradient, which is in agreement with the thermal distribution simulation; when the flow was applied, the image shows a distortion in the otherwise concentrated temperature profile of the heater (Figure 9a), caused by the convective effect of the liquid, which can be followed thought the flow direction of the microchannel (Figure 9b).

**Figure 10:**
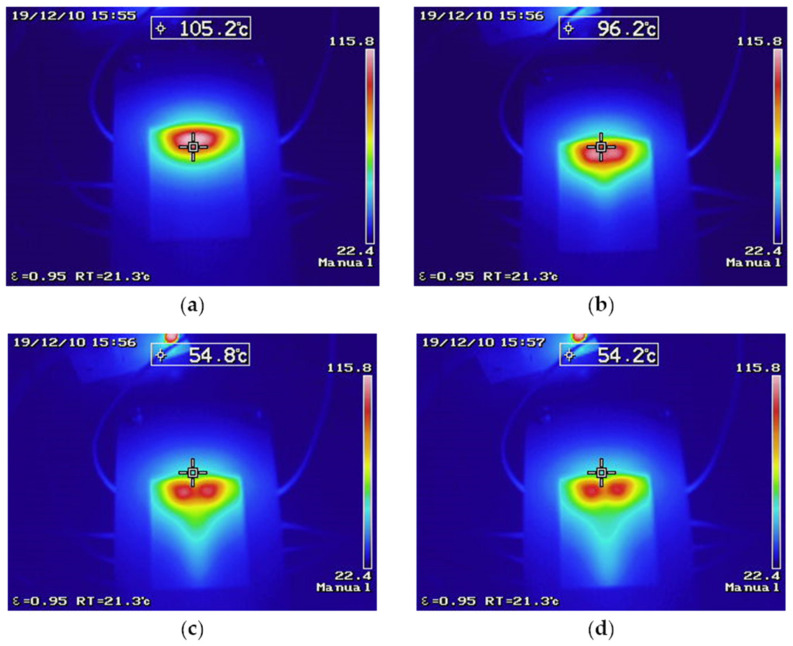
Thermographic camera results in testing with different flows to inspect the heat velocity phenomena: (**a**) 5 mL/h; (**b**) 10 mL/h; (**c**) 15 mL/h; (**d**) 20 mL/h.

To observe the effects of the heat transfer through convection on the heater surface, the microsystem was set to operate at a temperature of 115 °C. The flow rates used were 5 mL/h (Figure 10a), 10 mL/h (Figure 10b), 15 mL/h (Figure 10c), and 20 mL/h (Figure 10d). The influence of the flow rate on the contour of the heat profile can be noted for each case studied. While, in the first image capture, the heat profile distribution was seemingly unaffected by the flow of the ambient temperature liquid, for the next cases, the heat profile contour showed an elongation, determined by the flow direction, and of a longitude that could be adjusted as a function of the flow rate. The direct effect of the flow over the heater surface can also be noted. As the flow rate increased, the convective heat transfer was more evident, showing a decrease in the temperature recorded at the heater–microchannel intersection; this phenomenon was not observed at lower temperatures and can be related to the large temperature difference between the fluid and the heater surface, as well as the convective heat transfer in the fluid, which prevented the system from completely elevating the fluid temperature at the heater focal point. These test results led to the conclusion that the system allows keeping the temperature focused in the heating zone, evidencing the temperature flow, which can be used to control the temperature in different processes and to develop thermal flow sensors to monitor changes in the flow across a microsystem.

## 4. Discussion

The thermal convection loss integrated into the lab-on-chip fabricated device can be reduced, with an analysis of the contact resistance between the pads and the cables of the attached sensors and a better understanding on the heat transfer process. For instance, Jiang [12], Byers [21], and Tiwari [22] used a time response of the temperature for a specified provided voltage, which can be very useful to describe the transient thermal analysis [3]. In the work of Jiang [12], this temporal curve characterization with hysteresis was developed, showing the fluid heating response for a provided pulsating power between 0.2 W and 3.2 W, and a flow rate of 4 mL/min, demonstrating the rapid heat transfer process when a liquid flow is applied, which, although not considered in that work, provides a key characteristic for use in microsystem flow sensors.

The common theoretical resistance calculated in this work was less than the practical resistance measured with respect to the heater, which is related to the effects of resistivity on thin films that should be considered with a different model. In the model presented by Byers [21], a resistor performance analysis in terms of response time was used, which can be advantageous. An appropriate model can be used for materials that are reduced to dimensions on the nanoscale, in which many of the common properties or characteristics are no longer valid; likewise, it has been shown that the mechanical, thermodynamic, electrical, and optical properties are altered due to size differences. The reasons for this change in properties are related to increased surface interactions, as well as absorption and dispersion effects [32].

Diverse approaches have been used to date for fabricating microheaters; for example, Byers [22] and Tiwari [23] presented printed microheaters, in both cases, using COMSOL for modeling temperature and voltage distributions, which achieved temperatures up to 67 °C and 100 °C, respectively. In the case of PVD microheaters, Scorzoni [20] showed a similar method to the current work, using PVD to attain a Cr/Al/Cr resistive thin film. The experimental characterization used in that work presented a different linear behavior of the response curves in a measurement of the resistance of four terminals, for a temperature range of 40 °C to 90 °C, highlighting the benefits of serpentine shaped microheaters, as well as using simulation to calculate a more efficient geometry, changing the transverse direction, the linewidth, and the line-gaps, where these changes extend the temperature uniformity along the microheater. In this respect, as exposed by Wu [33], serpentine heaters are more flexible than spiral heaters in achieving heat surface uniformity and, thus, a larger temperature distribution area. These characteristics allow considering serpentine-based heaters as a good candidate for ultralow-power microheater devices [34], as well as for applications of fluid flow measurement.

Lastly, it can be observed that other materials, such as gold, used in a similar fashion to copper, with similar development techniques, reflect a better response time for resistance [23]. On the other hand, platinum, in the prospective characterization process for microheaters, may decrease the response time and allow for a more linear characterization curve, using a wider temperature range, as shown by Garraud who used a Pt layer with PVD techniques in a heater to maintain a 37 °C temperature [24], at the expense of increasing material costs for production. In comparison, the copper–chromium heater had a better temperature response to the power applied (71.3 °C for 0.7 W) than other materials such as aluminum in the microheater built by Nieto [25] over a soda–lime glass substrate covered with an aluminum layer with a thickness of 120 nm (71.3 °C for 0.94 W). Moreover, the current approach also introduced a microsystem integration with techniques that can be used outside of a clean room facility, which can be a desired characteristic for research in emerging countries.

## 5. Conclusions

A PMMA microfluidic system microsystem that incorporates a copper–chrome-based glass heater was designed, simulated, and characterized by a hysteresis process for a voltage range of 0 V to 3.2 V, corresponding to temperatures between 20 °C and 85 °C. The microsystem was built and integrated with a temperature control to use at the temperature reference point. The system was simulated for 2.47 V and a power of 0.67 W, with a resulting temperature of 69.8 °C, at a room temperature of 20 °C. The microsystem produced a temperature of 71.3 °C in measurements at a room temperature of ~21.3 °C, with a provided voltage of 2.53 V and a current of 279 mA (0.70 W of power).

The electrothermal results supported the simulation, with a 7.22% error for the heating characterization and a 5.42% error for the power consumption. Furthermore, the chosen reference temperature point backed up the distribution temperature and voltage, which concluded that the system can be used to control the temperature in different processes and to develop thermal flow sensors, with the advantage of having the flexibility of being coupled in different systems, as well as having optical transparency and repeatability in its fabrication, specifically in the vapor deposition process.

## Figures and Tables

**Table 1:** Properties used in the electrothermal simulation in COMSOL Multiphysics^TM^, with properties given by the material selected in the simulation.

Item	Value
Initial air temperature (°C)	20
Copper electrical conductivity (S/m)	5.96 × 10^7^
Chrome electrical conductivity (S/m)	7.9 × 10^7^
Silica glass electrical conductivity (S/m)	1 × 10^−14^
Copper thermal conductivity (W/m·K)	400
Chrome thermal conductivity (W/m·K)	93.7
Silica glass thermal conductivity (W/m·K)	1.38
Air heat transfer film coefficient (W/(m^2^·K)) [29]	20.45
Copper layer thickness (nm)	100
Chrome layer thickness (nm)	15

**Table 2:** Measurements and common theoretical calculations of the copper-chrome-based heater.

Item	Copper	Chrome	Heater
Height (nm)	100	15	115
Resistivity (Ω⋅mm^2^/m) [30]	0.017	0.125	-
Pad area (mm^2^)	0.00125	0.0001875	-
Pad length (mm)	7.43	7.43	-
Heater length (mm)	31	31	-
Path resistance (Ω)	0.101	4.9538	0.0989
Heater resistance (Ω)	5.301	38.75	4.66
Heater resultant resistance (Ω)	5.402	43.73	4.80

## References

[1] Khan B., Ahmed S., Kakkar V. (2016). A Comparative Analysis of Thermal Flow Sensing in Biomedical Applications. Int. J. Biomed. Eng. Sci..

[2] Das S., Kumar R., Singh J., Kumar M. (2020). Fabrication of Microsensor for Detection of Low-Concentration Formaldehyde Gas in Formalin-Treated Fish. IEEE Trans. Electron. Devices.

[3] Jung G., Hong Y., Hong S., Jang D., Jeong Y., Shin W., Park J., Kim D., Jeong C.B., Kim D.U. (2021). A low-power embedded poly-Si micro-heater for gas sensor platform based on a FET transducer and its application for NO2 sensing. Sens. Actuators B Chem..

[4] Paun C., Tomescu R., Cristea D., Ionescu O., Parvulescu C. Design, fabrication and caracterization of a micro-heater for metasurface-based gas sensors. Proceedings of the 2020 International Semiconductor Conference (CAS).

[5] Keshavaditya G., Eranna G.R., Eranna G. (2015). PRT embedded microheaters for optimum temperature distribution of air-suspended structures for gas sensor applications. IEEE Sens. J..

[6] Bagga S., Akhtar J., Mishra S. (2020). Preeti Fabrication of coplanar microheater platform for LPG sensing applications. Microsyst. Technol..

[7] Ricci P.P., Gregory O.J. (2021). Free-standing, thin-film sensors for the trace detection of explosives. Sci. Rep..

[8] Rezania A., Rosendahl L.A. (2012). Thermal effect of a thermoelectric generator on parallel microchannel heat sink. Energy.

[9] Hayakawa T., Sakuma S., Fukuhara T., Yokoyama Y., Arai F. (2014). A Single Cell Extraction Chip Using Vibration-Induced Whirling Flow and a Thermo-Responsive Gel Pattern. Micromachines.

[10] Hernandez C.A., Beni V., Osma J.F. (2019). Fully automated microsystem for unmediated electrochemical characterization, visualization and monitoring of bacteria on solid media; *E. coli* K-12: A case study. Biosensors.

[11] Velve-casquillas G., Le M., Piel M., Tran P.T. (2010). Microfluidic tools for cell biological research. Nano Today.

[12] Jiang L., Wong M., Zohar Y. (2000). Unsteady characteristics of a thermal microsystem. Sens. Actuators A Phys..

[13] Lagally E.T., Emrich C.A., Mathies R.A. (2001). Fully integrated PCR-capillary electrophoresis microsystem for DNA analysis. Lab Chip.

[14] Liu X., Li L., Mason A.J. Thermal Control Microsystem for Protein Characterization and Sensing. Proceedings of the 2009 IEEE Biomedical Circuits and Systems Conference.

[15] Tvarogek V., Tienb H.T., Novotny I. (1994). Thin-film microsystem applicable in (bio) chemical sensors. Sens. Actuators B Chem..

[16] Bermudez J.F., Saldarriaga J.F., Osma J.F. (2019). Portable and low-cost respirometric microsystem for the static and dynamic respirometry monitoring of compost. Sensors.

[17] Wojtas N., Hierold C. Microfluidic heat transfer systems optimized for thermoelectric heat exchangers. Proceedings of the 2013 Transducers & Eurosensors XXVII: The 17th International Conference on Solid-State Sensors, Actuators and Microsystems (TRANSDUCERS & EUROSENSORS XXVII).

[18] Pawlak R., Lebioda M. (2018). Electrical and thermal properties of heater-sensor microsystems patterned in TCO films for wide-range temperature applications from 15 K to 350 K. Sensors.

[19] Je J., Lee J. (2014). Design, Fabrication, and Characterization of Liquid Metal Microheaters. Microelectromech. Syst..

[20] Scorzoni A., Caputo D., Petrucci G., Placidi P., Zampolli S., de Cesare G., Tavernelli M., Nascetti A. (2015). Design and experimental characterization of thin film heaters on glass substrate for Lab-on-Chip applications. Sens. Actuators A Phys..

[21] Byers K.M., Lin L.K., Moehling T.J., Stanciu L., Linnes J.C. (2020). Versatile printed microheaters to enable low-power thermal control in paper diagnostics. Analyst.

[22] Tiwari S.K., Bhat S., Mahato K.K. (2018). Design and fabrication of screen printed microheater. Microsyst. Technol..

[23] Gregorini M., Grass R.N., Stark W.J. (2020). One-Step Photolithographic Surface Patterning of Nanometer-Thick Gold Surfaces by Using a Commercial DLP Projector and the Fabrication of a Microheater. Ind. Eng. Chem. Res..

[24] Garraud A., Basrour S., Peyrade D. Fabrication of a Multiple Heater-Sensor Platform for Cell Temperature Monitoring. Proceedings of the 2020 Symposium on Design, Test, Integration & Packaging of MEMS and MOEMS (DTIP).

[25] Nieto D., McGlynn P., de la Fuente M., Lopez-Lopez R., O’connor G.M. (2017). Laser microfabrication of a microheater chip for cell culture outside a cell incubator. Colloids Surf. B Biointerfaces.

[26] Aranguren A., Torres C.E., Muñoz-Camargo C., Osma J.F., Cruz J.C. (2020). Synthesis of Nanoscale Liposomes via Low-Cost Microfluidic Systems. Micromachines.

[27] Campaña A.L., Sotelo D.C., Oliva H.A., Aranguren A., Ornelas-Soto N., Cruz J.C., Osma J.F. (2020). Fabrication and characterization of a low-cost microfluidic system for the manufacture of alginate-lacasse microcapsules. Polymers.

[28] Perez M.A. (2014). Instrumentación Electrónica.

[29] Khabari A., Zenouzi M., Connor T.O., Rodas A. Natural and Forced Convective Heat Transfer Analysis of Nanostructured Surface. Proceedings of the World Congress on Engineering.

[30] Serway R.A., Jewett J.W. (2014). Physics for Scientists and Engineers with Modern Physics.

[31] Cadar S. Simulation & Modelling of a Tungsten Filament with COMSOL for Electrothermal Process. Proceedings of the 2016 IEEE 22nd International Symposium for Design and Technology in Electronic Packaging (SIITME).

[32] Lacy F. (2011). Developing a theoretical relationship between electrical resistivity, temperature, and film thickness for conductors. Nanoscale Res. Lett..

[33] Wu Y., Du X., Li Y., Tai H., Su Y. (2019). Optimization of temperature uniformity of a serpentine thin film heater by a two-dimensional approach. Microsyst. Technol..

[34] Mahdi M. (2021). Ultra-low power MEMS micro-heater device. Microsyst. Technol..

